# Industrial clusters, creating a strategy for continued success

**DOI:** 10.1016/j.heliyon.2024.e29220

**Published:** 2024-04-04

**Authors:** David McKernan, Olivia McDermott

**Affiliations:** College of Science & Engineering, University of Galway, Ireland

**Keywords:** Cluster, Medtech, Medical device, Ireland, Europe

## Abstract

**Purpose:**

This study aims to describe and propose an effective strategy for an industrial cluster and investigates industrial cluster-promoted growth.

Design/Methodology/Approach: A qualitative study was carried out within the Irish Medtech cluster with key stakeholders from the cluster.

**Findings:**

The findings demonstrate significant opportunities for further cluster development and to potentially double the number of spin-outs and value of R&D investments while enhancing an entrepreneurial culture. This study also recommends that multiple agencies collaborate to achieve the future strategic objectives of the cluster. The soft infrastructure of regulatory and intellectual policies is as important as the hard infrastructure of roads and buildings.

**Practical implications:**

This study has implications for government policy, informing how they align the local needs of compact industrial clusters with policies to make the Irish cluster more unique and gain a sustainable competitive advantage. The approach is applicable and has implications for all industrial clusters.

Originality/Value: This is one of the first studies to look at the Irish Medtech cluster in terms of its shortcomings and future opportunities.

## 1 Introduction

Medical devices are products, services, or solutions that prevent, diagnose, monitor, treat, and care for human beings by physical means. Medical devices are diverse, ranging from simple bandages to complex diagnostic imaging equipment or invasive cardiovascular stents [1]. Industrial clusters are essential to the competitive advantage of regions [2]. The clustering effect is significant, with 39% of European jobs and 55% of European wages located in clusters [3]. According to Porter [2], clusters are a source of highly localised strategic competitive advantage. The competitive advantage effect of a cluster is very “spiky” contained in small geographic areas of approximately 5–10 km in diameter [4]. Innovation is a crucial factor in a cluster, and it is reported to be responsible for 65% of productivity growth and 62% of the total growth in Europe [5]. Start-ups have several benefits to clusters by providing both differentiated innovation and high growth [6].

Ireland has had a strong medical device manufacturing cluster since the early ’90s [7]. This paper focuses on the medical device ecosystem in Galway, Ireland, the main actors in the system, their interactions, and how to improve the competitive advantage of the cluster. Most of the industry started when multinationals from the United States moved to Ireland in the early 1990s [8].

Multinationals have played a strong role in the cluster's growth, bringing jobs, innovative products, spin-off companies, and opportunities for graduate employment [9]. An ecosystem has developed with strong linkages between universities, start-ups, multinationals, venture capital, suppliers, and supporting industries. The Irish Medtech cluster has had increasing success over the past three decades, but with increased globalisation, cost of production, and other competitive clusters arising in, for example, Costa Rica, there are concerns about the future of the Irish cluster [6]. Ireland had successful clusters in electronics and computer manufacturing in the late 1970s and 1980s until the 1990s, which no longer exist due to outsourcing production to cheaper manufacturing locations globally [4,10].

Other medical device clusters have different characteristics from Ireland. Tuttlingen in Germany is an example of a specialist Medical Device cluster that has evolved organically over many years with a narrow specialisation in surgical instruments [11]. Medicon Valley is a cluster centered around Copenhagen, it has developed strong indigenous companies that originate in the 1800's [12]. Medicon valley has made the effort to brand itself as a location for medical and pharmaceutical expertise. Israel has created medical device cluster. The Israel cluster has grown from research, accelerators, and the availability of venture capital [13] Unlike Ireland the Israeli cluster has little manufacturing but has significant research and development.

Thus, this paper seeks to address the following research questions (RQ's).

RQ1 What are the current strengths and weaknesses of the Irish Medtech cluster in terms of having access to human capital, funding, innovative products and practices, demand for products, good infrastructure, and culture?

RQ2 What are the opportunities to improve the Irish Medtech cluster's health, culture, collaboration, and future regarding human capital, funding, innovative products and practices, demand for products, good infrastructure, and culture?

Section 2 contains the Literature Review; Section 3 outlines the qualitative methodology. Finally, the results are outlined in Section 4, while the discussion and conclusion are put forward in Sections 5, 6.

## 2 Irelands Medtech cluster – an overview

Ireland's medical device industry is dominated by multinationals that established manufacturing sites in the 1990s [9]. Ireland was able to attract the world's largest medical device companies due to its proximity and tariff-free access to the EU, relative cost competitiveness, English speaking, and responsive regulatory systems [14]. Ireland's low corporate tax rate (10% in the 1990s) is often quoted as a reason for FDI. While its acknowledged corporate tax rates play a role in FDI, McKernan and McDermott [14] suggested alternative reasons of skilled workforce, stability, membership of the EU and a regulatory regime conducive to doing business. Medical device firms self-reported the availability of skilled labour as the most important factor when selecting a site [15].

31% of the medical device companies in Ireland are in Galway and Galway's medical device start-up rate per head of population is ten times the average for Ireland [6,16] with Galway's local university having had 18 life science and medical device spin-outs.

**Fig. 1:**
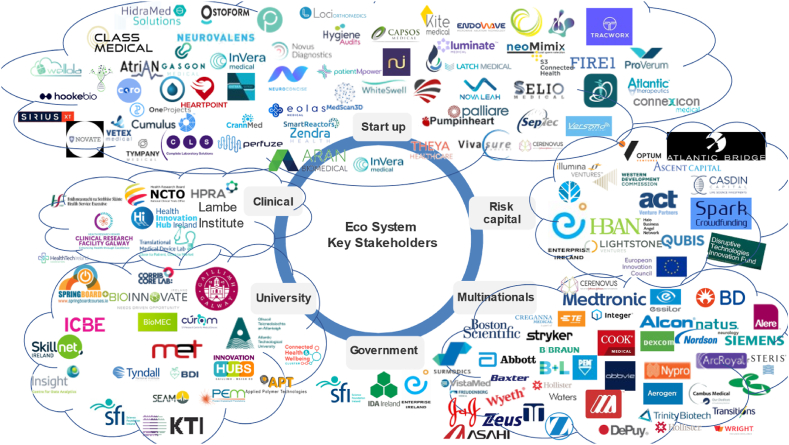
Key stakeholders in the Medtech cluster in Ireland (Source: Author's work).

The Galway city cluster was kick-started with multinationals moving to the city [10]. Location is important, with 83% of organisations stating that location is important in choosing a manufacturing site [16]. Studies have shown that being in a cluster generates output gains that are six times that of the extra costs [17]. Fig. 1 shows key stakeholders in the Irish medical device cluster. The key stakeholders in the Irish cluster are made up of multinationals, start-ups, clinical trial facilitators, venture capitalists, government agencies and universities [4]. It has been shown that the linkages between stakeholders are critical for cluster success [18].

Various success factors or elements in a Medtech cluster's success include having access to human capital, funding, innovative products and practices, demand for products, good infrastructure, and culture [19].

### 2.1 Innovation amongst medtech clusters

Innovation is the key driver of a cluster's competitiveness, productivity, economic health and the overall economy [20]. Ireland was ranked 23rd globally on the innovation index in 2022 and had been slipping down the rankings. Ireland scored highly in institutional measures but poorly in market sophistication [19]. Although the innovation index scores show Ireland's innovation ranking reducing over the last few years, the method inaccurately measures several key statistics for Ireland. Ireland's Gross Domestic Product (GDP) is significantly higher than its Gross National Product (GNP) [6,14]. Several of the sub-measures use GDP as a denominator. For example, general infrastructure has a sub-measure “Gross capital formation” as a % of GDP, and Ireland's scores are below Israel and Sweden using this metric. When Gross National Product (GNP) was used, Ireland's score increased from 20.8 to 29.7, placing it ahead of Israel and Sweden. Ireland's Innovation score thus is negatively affected due to its GNP being only 70% of its GDP (19).

Recent new medical device regulations (MDR) in Europe have meant that it is now more cumbersome to get a new innovative device to market in the European Union, and thus the United States is now being seen as the first market of choice in terms of launching a new device [1,21]. Such Regulatory restrictions will stifle innovation in Europe and potentially result in slower growth of new product manufacturing.

### 2.2 Human capital

Human capital is an important factor in driving successful organisations in clusters as it enables stronger clusters that improve regional employment and revenue growth over time and the resilience of regional economies to downturns [22].

A successful cluster relies on a strong local human capital base and a high level of inter- organisational employment mobility enables knowledge transfer [23]. [24] discuss how graduates and universities can play a key role in the medical device industry regarding human capital education, training, and research.

A recent study by McKernan and McDermott [4] on the Irish Medtech cluster's evolution and growth discussed the proven importance of the local University of Galway to its Medtech cluster success and the importance of Massachusetts Institute of Technology (MIT) to the Boston Medtech cluster.

Between 2008 and 2017, employment in medical device manufacturing in Ireland increased by 34% [25]. Since moving here 40 years ago, the multinationals have created a pool of skilled labour and, by working with the universities to create courses to meet these demands, have met their skills needs.

The local Irish higher education institutes (most recently designated as Technological Universities) and universities throughout Ireland have also provided a steady stream of graduates for the medical device industry. Courses have been specifically adapted for the medical device industry, including degrees and masters in Biomedical engineering and Biomedical science [4]. Supporting this, manufacturing and engineering apprenticeships have been developed with the help of newly designated regional Technological Universities (formerly Regional Institutes of Technology. As a result, Ireland has the highest proportion of graduates in Science, Technology, Engineering, and Mathematics (STEM) subjects in the EU, thus strengthening the human capital within the MedTech cluster.

### 2.3 Funding

**Fig. 2:**
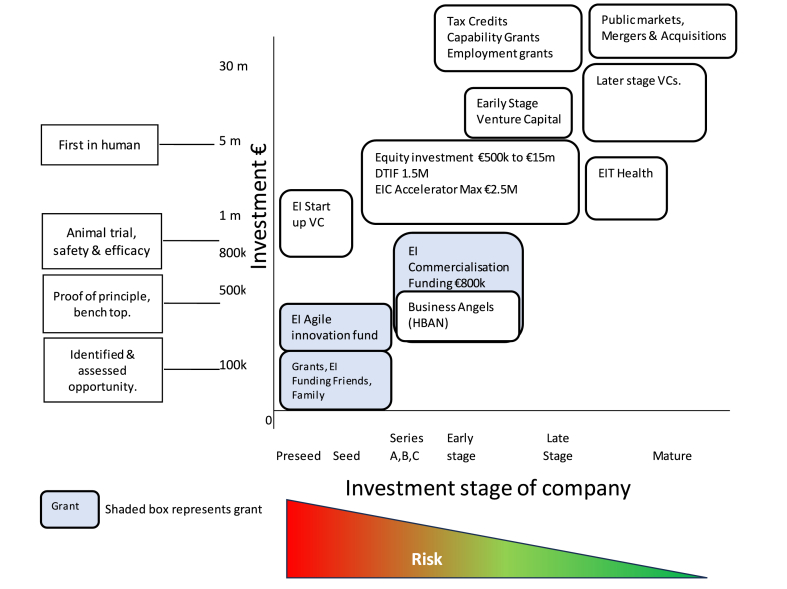
Investment options at different stages of a company (Source authors own).

Many researchers have emphasised the positive impact of venture capitalists' VC industries on clusters' growth, strength and success [4]. However, venture Capital (VC) and Research and Development (R&D) investment in Ireland is below that of competing nations. Total VC investment in Ireland accounted for 0.106% of Gross National Income (GNI). This is just 20% of the rate in the USA [26]. This is a concern as VC funding provides positive input that cannot just be obtained through public finances [27]. There are many different methods of funding innovation and start-ups globally, specifically in Europe and Ireland. Fig. 2 details typical funding sources utilised by MedTech companies in the cluster based on the stage of the company and the investment level required. Grants are available through Enterprise Ireland (EI) at early stages of the company when the risk is greatest.

The milestones on the vertical axis in Fig. 2 are inflection points that increase the valuation of the company (P5). For example, successfully completing an animal study reduces risk and significantly increases the value of the company. EI is the largest venture capital (VC) investor in Europe as measured by number of investments [28]. Its investments have been leveraged to generate 3.7 times more funding from commercial sources.

**Fig. 3:**
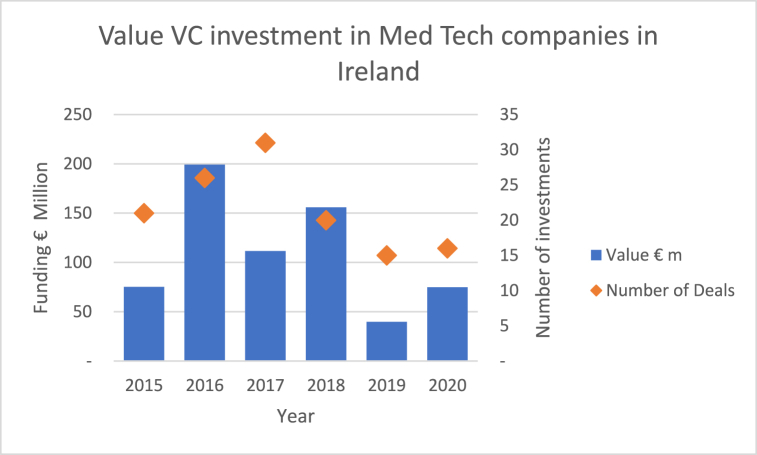
Venture Capital activity in Ireland for Medical Device companies (Author's own work based on data gathered from Irish Venture Capital Association).

Fig. 3 shows the VC activity in Ireland for medical device companies based on data from the Irish Venture Capital Association (IVCA) [29]. VC investments for non-medical device companies have been removed from the data.

**Fig. 4:**
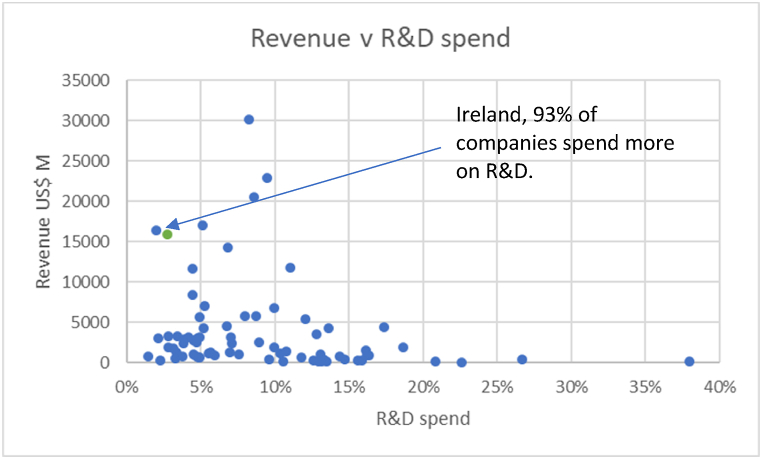
Ireland's R&D spend in relation to top 100 companies.

Research and development (R&D) spending in Ireland, is US$345 m in 2020. This equates to 2.8% of total export value for the year, is proportionally smaller than the industry average of 9%. This is despite “*the cost of R&D engineering in Ireland being approximately half that of the United States, and accessing talent are easier in Ireland*. *R&D is about confidence in the location. In multinationals, R&D is held tightly in central locations and is only moved to other locations when trust is built”* (**P11**). The annual revenue for the top 100 medical device companies globally was obtained from Medical Design and Outsourcing [30]. 74 of the top 100 medical device companies provided their global R&D spend. Fig. 4 plots the revenue and R&D spend of medical device companies in Ireland compared to global medical device companies. 93% of companies spend more as a percentage of revenue than Ireland on R&D. The figures are based on gross revenue from officially published data.

#### 2.3.1 Infrastructure

Many studies have highlighted the importance of good infrastructure in developing a cluster and fostering economic growth. Public investment in infrastructure is quite productive; a rich body of research has contributed to the establishment of a statistical linkage between infrastructure and economic growth [31,32].

Physical infrastructure, such as the availability of accelerators, has positively affected start-ups [33]. Soft infrastructure, such as regulations, significantly impact medical devices; for example, recent Medical Device Regulations (MDR) have increased the time it takes to release a product in the European market [6,34]. The medical device industry in Ireland is dominated by multinationals from the United States of America (USA) [9]. Multinationals have been shown to anchor the industry and increase resilience and innovation in a cluster [35].

### 2.4 Demand

Global demographics create a relentless increase in healthcare spending and growing global demand for Medical Devices. Healthcare spending increases by an average factor of 6 for those aged over 85 compared to those between 55 and 59 years and in addition, there are 728 million people aged 65 or over globally, projected to double to 1.5 billion in 2050.

The availability of novel medical devices to treat unmet clinical also creates new demand for medical devices. An example of new demand is Endovascular Stroke Therapy (EST). Saber et al. [36] showed a continuous increase in EST over the 10 years from 2006 to 2016. The number of ESTs went from less than 50 in 2006 to 1000 in 2016. Healthcare spending has more than doubled between 2000 and 2019, at US$ 8.5 trillion or 9.8% of global GDP [6].

The USA is the largest producer and consumer of medical devices [1]. Although the medical device market is continually growing, the price for undifferentiated products reduces yearly. Purchasing departments are making increasingly purchasing decisions with physicians in an advisory role. In the USA, hospitals are pooling their purchasing power to reduce costs. Between 72 and 80 per cent of non-labour purchases are completed through a Group Purchasing Organisation (GPOs) or Integrated Delivery Networks (IDNs) [37]. Centralised purchasing results in the standardisation of medical devices and downward price pressure. For example, the Average Selling Price (ASP) for a carotid balloon catheter is expected to drop by 26% from US$240 to US$178.90 over the 10 years from 2017 to 2027 (37).

### 2.5 Culture

The collective identity of the cluster is part of a soft infrastructure that sets behavioural norms and enables firms to create joint strategies. The collective identity can form part of the cluster's competitiveness [23]. Creating a competitive advantage is a highly localised process; values, culture, structures, institutions, and history are part of that process [2].

**Fig. 5:**
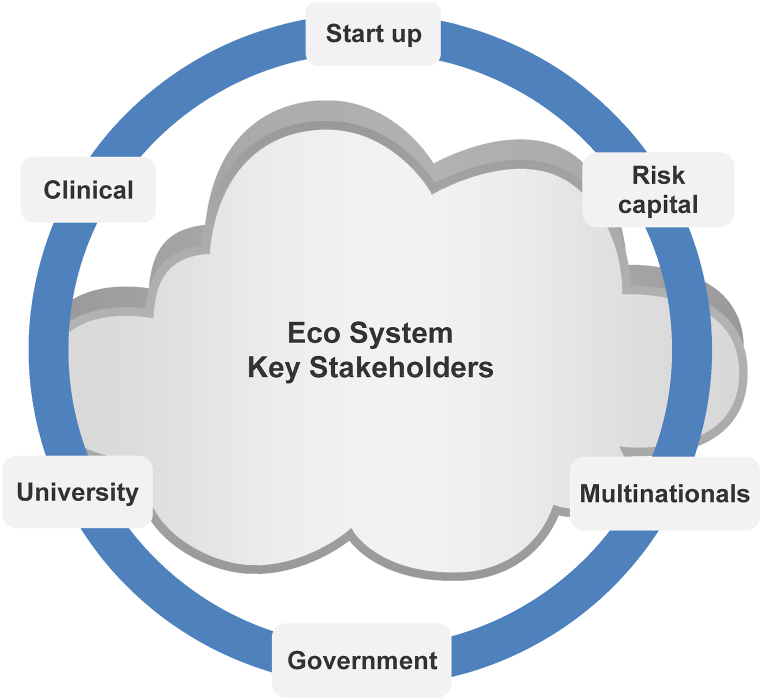
Firms and organisations in the ecosystem (Source: Authors own).

Innovation and a thriving regional economy depend on relationships and social networks between firms and organisations [38]. Fig. 5 shows the key stakeholder groups analysed as part of the ecosystem.

### 2.6 Conclusion

The medical device industry in Ireland has been extraordinarily successful. The cluster in Galway has been particularly successful and concentrated. A growing global market has helped the industry, but decreasing product prices is challenging for all medical device companies. In addition, changing regulations have created longer lead times to release products, slowing innovation.

## 3 Methodology

The methodology for this study includes qualitative data collection from interviews based on purposive samplings [39]. Senior managers in the medical device industry and key actors with knowledge of the medical device ecosystem were chosen for this study. Purposive sampling was utilised as it allows the researcher's judgment to select people, organisations, regions, and case studies to review [40].

A key purpose of the research is to determine the relative strengths of the Irish ecosystem and the linkages between stakeholders. To ensure a heterogeneous sample, representatives from each Medtech stakeholder group were interviewed, e.g., entrepreneurs, academics, government representatives, and members of the leadership team of multinational corporations. Based on literature [6,9,40] key stakeholders in a medical device ecosystem are Start-ups, risk capital, government, multinationals, government, universities and clinical. A set of fifteen questions concerning the elements of a Medtech cluster ecosystem were devised concerning these stakeholders. Questions were pre-planned but somewhat open and unstructured so they could be adapted for each stakeholder [41]. The list of questions is outlined in Table 1.

**Table 1:** Interview questions.

Themes	Questions (non-demographic)	Literature sources
Human Capital	1 What linkages and help have you had with higher education institutions? 2 Can more be done to translate basic research from universities to commercial ideas? 3 What is higher education works well and positively supports the medical device industry? 4 What has been your experience with how higher education institutions approach Intellectual property?	[4] [6] [42]
Funding	5 From your experience, is access to funding for start-up companies difficult? 6 Do venture capital companies demand too much equity? 7 Describe your experiences with government funding and grants? 8 Are there any stages in funding that are particularly difficult?	[6,10,14,42]
Demand	9 Are there medical needs that should be exploited/explored to grow the medical device cluster? 10 Are there services or critical supplies that we should establish in the ecosystem?	[6] [43]
Infrastructure	11 What physical things would help develop the medical device cluster? 12 Are there critical suppliers missing from the ecosystem?	[9,32,42]
Culture & Incentives	13 How would you describe the culture in the medical device ecosystem? 14 Is there regular collaboration in the ecosystem? 15 Are there regular events and locations where ecosystem members meet and collaborate?	[4,42]

The study took place over a 12-week period of interviews. The participant's contacts were obtained through researchers' knowledge within the industry and LinkedIn. LinkedIn was used as it is a commonly used networking site for professionals. The participants were selected or approached based on their experience and pedigree in terms of their knowledge of the Medtech sector. A selection of entrepreneurs in the Medtech sector, industry R&D professionals, researchers, CEO's, Founders of Medtech start-ups were all approached to achieve a broad representation of Medtech stakeholder across the Irish Medtech industry.

Participants were called or sent personalised emails explaining the purpose of the study and requesting their participation, and upon agreement, an online interview was organised. The interview started with questions on the participant's experience and background [44]. They then answered the pre-planned open-ended questions. The interviews and quotes are recorded verbatim, and everyone is given a participant number (P number) to maintain anonymity [45]. Eighteen people agreed to be interviewed and sixteen people were interviewed initially but interviews were stopped after the 16th interview as no new themes emerged after the 13th -interview and between the 16th interviews due to the data becoming saturated [46]. The participant summary details are outlined in Table 2.

**Table 2:** Participant information by position/stakeholder group.

Participant Number	Role	Stakeholder Represented	Experience	Comments
P1	Professor of Engineering	University	30 Years +	Also established several companies (non-medical devices).
P2	Leadership role in Technology transfer office	University	20 years +	University innovation hub
P3	Executive	Multinational	30 Years +	American multinational
P4	Business Consultant life sciences	Government	30 Years +	Innovation Hub funding
P5	Early-stage Entrepreneur	Start-up	15 Years	Start-up medical device company
P6	Founder/CEO	Entrepreneur	30 years +	Multiple spins out successfully raised several million in funding and the current CEO.
P7	Founder/CEO	Entrepreneur	30 Years +	Founder and successfully sold a medical device company.
P8	Engineer	University	5 Years	Recently graduated with PhD from Science Foundation Ireland “Cúram” research group.
P9	CEO/Founder	Entrepreneur	30 Years +	Management buyout software company for connected medical devices.
P10	CEO/Founder	Entrepreneur	30 years plus	Founder and investor in med tech companies.
P11	Director of Research and Development	Multinational	30 years plus	Multisite role
P12	Director of Research and Development	Multinational	20 Years	Successful start-up now owned by Multinational
P13	VP Scientific Affairs	Clinical	30 Years	Worked in several multinationals internationally.
P14	Chief Financial Officer	Funding	30 Years	Organised funding rounds for 3 start-ups
P15	Business Manager, Research Centre	University	20 Years	Research centre based in the university.
P16	Chief Technology Officer	Entrepreneur	35 years +	Serial Entrepreneur. Successfully started and sold multiple start-ups.

### 3.1 Data analysis of the interviews

**Fig. 6:**
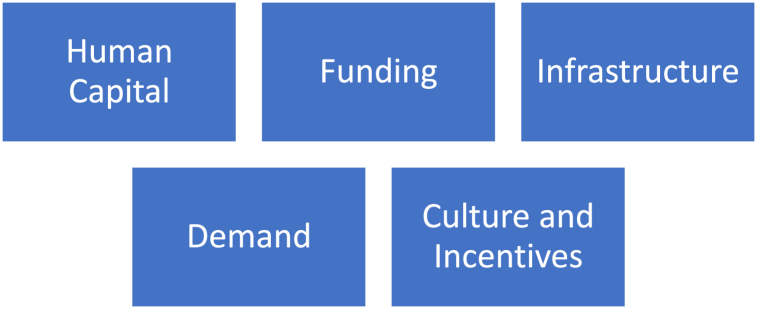
Thematic analysis plan.

The interviews were transcribed, identified, and uploaded to Atlas Ti9 software for qualitative analysis using the p numbers to maintain the anonymity of the interviewees. The answers were classified under different cultural, human capital, demand, infrastructure, and funding themes. Finally, the analysis plan of the study is depicted to give the reader an overview of the thematic analysis based on 5 thematic areas (Fig. 6).

Open coding was carried out to allow the different thematic areas to be linked [47]. Open coding was used to identify individual units, and axial coding was utilised to select major themes. The memoing and the member-checking technique aided the researchers in keeping track of the research themes while coding with two researchers verifying data. Inter-rater reliability was calculated to obtain consensus on the various questions related to the 15 interview sub-questions and was found to be 97%, deemed a satisfactory rating [48]. Any differences in thematic categories were settled through discussion between coders.

While a limitation of the study could be that there were only 16 experts interviewed, the interviewees were representative of the wider Medtech stakeholder and support environment. Qualitative research, by nature, can be biased, and the interpretation of qualitative data is made subjective [49]. Bias was minimised by detailed record keeping, ensuring coding and memoing were consistent and transparent; reviewing interviewee accounts was critical to understanding similarities and differences of perspectives [44,50]. Lastly, capturing detailed verbatim descriptions of interviewee accounts supports findings [51].

## 4 Results

The thematic areas that contribute to the success of the clusters are analysed based on the interview's answers.

### 4.1 Human capital

To assess how the availability of skilled labour and the Medtech industries' interactions with higher education institutions has aided the cluster's success, participants were asked about linkages with higher education institutions. These questions included how basic research was translated from universities to commercial ideas. They were also asked what in higher education works well and positively supports the medical device industry and their experiences with how higher education institutions approach Intellectual property (IP). A sample of responses concerning these questions is highlighted in Table 3.

**Table 3:** Interviewee Comments in relation to Human capital questions.

“*Universities are doing the right things; it is just that the capacity is not keeping up with growth in the industry*” (P7).
“*Universities are a huge untapped asset. More entrepreneurial training is required. We need to encourage innovation. Universities can create new businesses where there is no seed to begin*” **(P6)**.
“*Our Irish medical device company was started by a professor who wanted jobs for graduates in the late 80s*” **(P9)**.
“*Trying to start commercial ventures is difficult with small rewards. Culturally there is little prestige or glory in an academic trying to start a commercial activity. We need to improve the permeability of academia*” **(P6)**.
“*The outcomes are considered a best-in-class example of innovation in universities. Also, at the “basic coal face of manufacturing,” they were very good with technical challenges*” **(P7)**
“*In Ireland, we have almost unrestricted access to universities. If you want to talk to Stanford, you end up with a lawyer in the room. In Ireland, you can drop in and discuss what research is being carried out*” **(P10)**.
“*One concern with higher-level institutions is their attempts to claim Intellectual Property (IP) for small research projects where some materials need to be tested or checked. Universities should not be claiming for IP. The structure and agreement should be simple and agreed upon nationally. Some universities make it difficult to do Research and Development (R&D) because the terms are too onerous*.” **(P10)**.
“*It is expensive to get consultants to advise and help start-ups. They have been charged €2k a day” to provide advice for start-ups* “**(P2)**.
“*Ireland's skills base is weak in marketing sales and reimbursement*” **(P6)**.
“*There is a lot of unencumbered IP sitting on University shelves, but few have come from the outside to leverage it*” **(P1)**
“*If successful Universities expect to recoup the IP perhaps at 1.2 to 1.5 times the cost of research.*” **(P15)**
“*Start-ups cannot afford to pay experienced staff. They hire young engineers who are attracted to city lifestyle and the values of the company*” **(P4)**
“*We are short expertise in digital technology in medical devices”***(P4)**

Entrepreneurs spoke very positively about the impact of the Bio-Innovate program. Entrepreneurs have concern about universities owning IP is supported by academic literature. For example, it has been shown that a 10% decrease in the universities’ equity stake in a company leads to an estimated 3% increase in the probability of raising venture capital and an 8% increase in the number of spin-outs [52].

The Technology Transfer Office (TTO) in the university “*acts like a middleman to enable research ideas to be put to commercial use*, *the TTO's do not make money, and Galway is not set up to make money; it provides a service between research and scaling a company, the TTO acts like a middleman to enable research ideas to be put to commercial use*” **(P2)**. Commercial returns from university research are poor, even in world-class universities. For example, John Hopkins University spent US$ 1.5 billion on research in 2012 and produced US$16 million in licensing fees, which is approximately a 1% return. When expenses were considered, Stanford University had less than a 1% return on US$1.7 billion in research. The terms that universities request for IP have an impact on entrepreneurial activity. For example, a 10% decrease in university stakes leads to a 3% increase in VC funding and an 8% increase in university spin-outs [52].

### 4.2 Funding

To assess the methods of obtaining funding and the nature of funding used in the medical device industry, questions were asked about the interviewee's experience in accessing funding from venture capitalists, government funding and grants and if some funding stages are more difficult than others.

There are a range of options by which start-ups can access a range of grants and funding. However, “*a nightmare*” summed up several entrepreneurs' views on getting funding. In one example, the entrepreneurs had “*15 different classes of share in their business. This makes management of the business and selling the business complex as in Ireland, there is a gap between seed rounds and series A*” **(P7)**. Another interviewee stated that “*Ireland badly needs another 1 or 2 seed investing organisations*. *There is a shortage of VC* funds *that will provide seed* funding” **(P16**).

A second entrepreneur stated that they raised “*all their* funds *abroad*”. They stated there had been a “*dramatic improvement*” **(P16)** in the Irish Venture Capital (VC) industry over the last 10 years. “*There was* 1 *V C* fund *in Ireland in 1996, and now there is a network of them*” **(P16)**. However, “*bad terms can destroy a company; VCs want to capture the upside and hence create terms that discourage growth*” **(P6)**. Another participant commented, “*VCs will not invest in early-stage companies. They expect first in human trials to be completed and patents at least applied for*” **(P10)**.

For mature companies with revenue streams, the process is more positive. One CEO stated they are “*continually approached by private equity firms offering money to them*.” They elaborated, “*Start-ups in the USA can raise significantly larger levels of* funds*, and it is not unusual to have USA start-ups that have raised US$ 40 M as venture capitalists in the USA are much more willing to take significant risks*”**(P9)**.

“*The University of Galway Technology Transfer Office encourages academics to move research into commercial use by linking the research to grant funding*” **(P2)**. Government directly supports start-ups through Enterprise Ireland (EI). The extent of the investment can be seen because EI has become the largest seed investor in Europe (77). However, “*while EI is seen as a good source of funds, they are highly bureaucratic*” **(P2)**. One multinational stated that “*they did not want to work with a company funded through Enterprise Ireland again as it was too much work and administration*"**(P3)**. Surveys show that 57% of companies said the red tape associated with grants puts them off applying for them (78). EI funding, however, is important for the Irish Medtech cluster, as surveys show that 80% of start-ups find getting private capital difficult or very difficult.

EI provides grants for the early stage of a company when risks are highest. A high-potential start-up can get a commercial grant of up to €800k in return for 10% equity in the company, which gives the start-up an instant valuation of €8 M. On discussion of the grants available it was stated “*We are living in a golden age of* grants” (P5). “*However, Irish state rules mean that EI is not allowed to own more than 10% of the company. The* funding *is designed to get the product to animal trials and secure initial Intellectual Property (IP)*” **(P5)**. Thus, funding may not cover the full amount required to get the product to be launched. Another participant highlighted*, “It is important to talk with VCs at least a year before commercialisation* funding *runs out* -*once that* funding *is spent, it is like getting thrown out of the little bubble*” **(P4)**.

The high initial valuation that EI puts on the company has some unintended consequences, as “*for later-stage potential investors, the high valuation can make any transaction financially unjustifiable*” **(P10)**. They further elaborated that “*from concept to sale can take 9 years as the costs are “off the charts” and early investors will have their investments diluted quickly*” **(P10)**. Also “*Convertible loan notes allow investors to invest but convert to share at valuations set in future funding rounds. This avoids significant dilution*” **(P16)**.

In general, the participants see funding from EI as positive, but the types of shares they demand were seen as an issue “*as EI require convertible redeemable preference shares as a condition of* funding *and the share also has a coupon (Interest rate)*. *In addition, in the event of the company closing, EI shares have first preference over the remaining assets*” **(P7)**. EI was also seen as “*slow*” by another company and “*not moving at the speed of the VCs*” **(P14)**.

Venture capitalists are another source of funding for Medtech companies; however, they, too, had their challenges to deal with. “*Aside from EI, other investors in Ireland will not invest until a mini-clinical trial is done, so Start-ups need to use an accelerator or reach out to USA venture* funds*. Irish VCs are saturated with investments in medical devices, and the Irish investment community devalues all our start-ups”* **(P5)**. This can significantly impact start-ups as VCs have been shown to improve innovation, growth, and sales of start-ups [53]. The key benefits apart from the funding of a VC are coaching and adding credibility to the start-up. An entrepreneur did feel that “*the VCs are excellent at selecting companies with a large potential upside*” **(P10)**.

Angel investors in Ireland are important with one interviewee stating that *“the Halo Business Angel Network (HBAN) is useful in providing* funding” **(P4)**. Another elaborated that “*VCs will follow the smart money; if the right angel invests, the VCs will follow*” **(P16).** Other avenues used are “*the Disruptive Technology Innovation* Fund *(DTIF), European Innovation and Technology, and the European Innovation Council (EIC) with up to €2.5 m available non-diluting …. are some sources of* grants*. However, the* grants *are administratively heavy and difficult to apply for”* **(P4)**. The DTIF will fund a minimum of €1.5 m over 3 years and requires at least 3 independent partners.

Another interviewee discussed how “*some local companies have used crowdfunding*” **(P4)**. An example of crowdfunding is Auri Gen Medical which raised €2.3 m in December 2022 (82). “*Local multinationals would be prepared to invest in start-ups, but the* funding *is generally controlled at headquarters on a site out of the country* “**(P2)**. There can be a mismatch in expectations, and a typical crowdfund investor wants their money back in one or two years. One entrepreneur reiterated the comments and stated he “*felt that multinationals should do more corporate venturing, creating small* funds *to encourage accelerators*” **(P6)**.

**Fig. 7:**
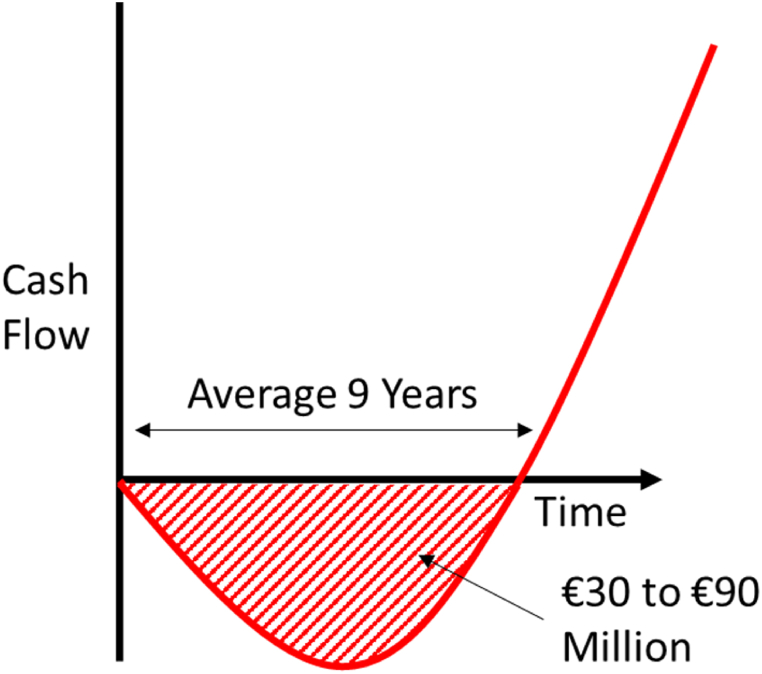
Typical cash flow/time for a medical device start-up (Source: Authors own based on interview data).

The comparison of Ireland's funding mechanism with other regions was a recurring theme. Israel was cited as an example where “*seed money is readily available*” **(P2)**. The government in Israel can play a more direct role as they are outside the EU, and unfair competition rules do not apply **(P16)**. The USA was cited as “*It is not unusual for a USA-based medical device start-up to raise US$ 50 to US$ 80 million. Irish start-ups will be drip-fed the venture money* “**(P9)**. Another participant elaborated*, “The initial* funding *in Ireland is seen as excellent, there is plenty of money in the early stages (lots of* grants*), and the issue is raising* funds *in the later stages”* **(P10)**. “*a medical device company typically needs to raise between €30 m to €90 m in Series B and C rounds to* fund *clinical studies*” **(P14)**. Fig. 7 shows the typical cash flow for a start-up medical device company.

Medical device start-ups are typical of an Innovation Driven Enterprise (IDE). IDEs are characterised by requiring significant funding, having substantial risk, selling to global markets, having a competitive advantage based on innovation and seeking to protect the innovation (82).

### 4.3 Infrastructure

To assess the infrastructural requirements for the ecosystem, the interviewees were asked what they thought could help develop the medical device cluster and if they thought that critical suppliers were missing from the ecosystem.

Many responded that the physical infrastructure for start-ups could be improved. “*We do not have space to host spin-outs*” at the university **(P2)**, and “the start-ups “*would accept a space in the toilet if they could get it*” **(P2)**. There are accelerators currently in place which “*are not good enough for medical devices as medical device companies need wet labs*” **(P2)**. To start, “*You need to get your hands dirty for innovation*,” which means building products in a clean room **(P6)**.

Others elaborated on the space theme stating that “*there is nowhere near enough room in the innovation hubs, “I Hubs” in Galway just doubled in size and must double again. Start-ups rent office space and turn it into clean rooms”* **(P16)**.

Clinical trials are important for start-ups, and this was another theme raised by interviewees. One interviewee stated, “*Clinical trials infrastructure in Ireland is terrible as the small population on the island makes it difficult to recruit patients for the trial*” **(P16)**. Other small countries are more successful than Ireland in recruiting patients and completing trials; for example, Denmark had almost 3 times the number of trials than Ireland between 2013 and 2020 (83). Clinical trials in Ireland are slow to start and often fail to hit their recruitment targets. 71% of trials fail to enroll the first patient within 180 days of the first submission to the Recognized Ethics Committee (REC)(83).

The time to approval for a medical device in Europe has increased from 9 months to 18 months due to regulation changes [14]. The Food and Drug Administration (FDA) in the USA has introduced a range of policies to enable patients to access new medical device technologies quickly. The policies for fast access include humanitarian device exemption (HDE), De Novo pathways, Emergency Use Authorization (EUA), and the Breakthrough Devices Program. Novel devices address an unmet need or can be a safer alternative to current devices. Between January 2018 and December 2020, 1090 manufacturers adopted the novel approaches to start clinical trials in the USA [54](.

Comments from the interviewees related to Infrastructure are outlined in Table 4.

**Table 4:** Interviewees comments related to Infrastructure.

*“Currently, we connect promising companies to Northwell in the USA to help with trials, and other Entrepreneurs tell us that they go internationally for clinical trials … In Ireland, the start-ups are too stretched for time, and Clinical access is critical; it would be fantastic if Ireland had better clinical trial access. Ireland is a great testing area as it has private and public hospitals, but it does not work for clinical trials”* (P2)
“*It is an advantage to have clinical trials near engineering centres*” **(P11)**.
“*Most clinical trials are in the USA as it is easier to get approval there*” **(P12)**
“*Ireland is known well for medical device technology. It is a pity there are no clinical trials in their own country*” **(P13)**.
“*An ideal clinical trial has quick access to an ethical committee. Ideally, the committee would approve a start-up's use on a case-by-case basis. This would enable fast feedback. A clinical site needs to have a dedicated area for therapeutic treatment. Staff should be trained in the relevant regulations of the International Council for Harmonization and Good Clinical Practice and ISO 14155. Dedicated resources are required for clinical studies. Patient follow-up is essential. Decentralised follow-up is the gold standard. Sending trained people to the patient's homes to complete clinically relevant tests makes it easy to have patients complete the study. Healthy patients tend to drop out of studies making the study's results look worse than they are*” **(P13)**.
“*Clinical trials are difficult in Ireland” We planned a trial in Dublin but had enrolled 100 patients in the USA before there was 1 registered in Dublin*”. **(P16)**
“*The HPRA (Health Products Regulatory Authority) are difficult to deal with; they ask questions that make it clear they do not understand the treatment area*” **(P16).**
“*Early development needs non-Good Laboratory Practice (GLP) animal trials. The centres require a Vet, Anesthetist, and physician to do the implant. Currently, these studies are done abroad. It is important to be able to do quick trials to learn about the product. In addition, the trials provide evidence of safety, which could include performance and handling*” **(P12)**.
“*Local access to animal and clinical trials is invaluable. We need flexible access. If you try a product that goes wrong, the team needs to re-engineer and test it again. This rapid prototyping is invaluable*” **(P5).**
“*We should build a large animal centre to do trials. A veterinary college would be ideal*” **(P16).**
“*Bio Excel was designed to be an accelerator. Unfortunately, no accelerator in place is suitable for Med Tech in Ireland. An example of world-class is J Labs. J Labs have ∼10 hospitals within* 2 km *of the lab; the access to hospitals makes J Labs different*” **(P2)**.

### 4.4 Demand

To assess market opportunities, the interviewees were asked what medical needs should be exploited or explored to grow the medical device cluster and if there were services or critical supplies that Ireland should establish in the ecosystem.

Ireland is 1.2% of the European Union population, the home market is small and hence new medical device companies must be “Born global”. The USA is the largest medical device market at US$ 225 billion, followed by the EU at US$128 billion and then China at US$61 billion (86). The comments from the interviewees are outlined in Table 5. There was a theme of regulatory difficulties delaying the introduction of new products. The recent stringent new European medical Device Regulations have led many companies deciding to launch in the US first [34].

**Table 5:** Interviewees comments related to Demand for MedTech devices.

*“In Ireland, the market is so small that we must start globally from day one. This means all the costs of a global business at the start. The EU is not a single market for Medical Devices. The CE mark is just the start. The bureaucracy of the EU makes it difficult to sell in. You must make different arrangements in each country to agree on pricing”****(P8)****.*
*“New products must be differentiated and fill a medical need not met in the market, like a tri-cuspid valve that can be implanted through minimally invasive techniques. I have seen many engineers say they have an idea for a better balloon or guide wire, but this will never work commercially”****(P10)****.*
*“I do not think I could develop my company today as I did in the past. EU rules are making it even more difficult. The costs of clinical trials are too much. In addition, pharmaceutical requirements are being pushed into medical devices”****(P11)****.*

Multinationals’ presence created a demand for companies to supply components, materials, and services **(P7), (P10)**. As a result, the medical device industry in Ireland is a global one. One interviewee stated that “t*here is significant potential in looking at people who are well and what we can do to prevent them from becoming ill. Potential new areas where the industry can create demand include digital services that link the patient, device, and clinician*” **(P9)**. Another stated “t*he trend is a movement out of hospitals; anything that prevents an overnight stay or hospital visit will have demand. In addition, any device that allows a procedure to be completed in an office or clinic or lets the patient be treated at home will have demand. Examples are remote monitoring or dialysis treatment at home”* **(P16)**. However another interviewees stated despite the demand for faster and more innovated treatments that “*reimbursement for digital solutions is poor. Clinical adoption is just not happening*” **(P4**).

### 4.5 Culture and incentives

To assess the culture in the Irish Medtech cluster, questions were asked as to how the interviewees would describe the culture and state of collaboration in the medical device ecosystem and if there were opportunities to meet and collaborate. The responses are summarised in Table 6. Generally, there was a very positive culture cited and one of camaraderie and collaboration for the overall good.

**Table 6:** Responses from the interviewees in relation to Culture.

“*Most informal connections happen at conferences or off-site gatherings*” (P4).
“*Leaders must have a long-term commitment to the ecosystem*” **(P1)**.
“*In universities, going commercial is difficult with small rewards. The lecturer is no longer an expert. There are few rewards and incentives that encourage translating research into commercial success*” **(P6)**.
“*It's a tight knit community; I can call my direct competitor and ask for help or advice*” **(P7)**.
“*Help and advice are only one phone call away*” **(P9)**.
“*The culture is supportive. Anyone who has been around the block actively helps. They are generous with their time to support start-ups*.” **(P16)**
“*Collaboration between institutions only happens at the optics level*” **(P4)**

Some more negative aspects of the culture were for example a researcher who had just completed a PhD focused on medical devices stated, “*that published papers were the only measure that counted and that they were unaware of any training to make ideas commercial*” **(P8)**. There was a lack of focus on entrepreneurship rather academic output was prioritized.

Multinationals could provide expertise to start ups as part of a panel of advisors. One interviewee stated, “*multinationals here in Ireland are willing to help others … indigenous firms with* funding*, but their own* funding *is from the external parent site*” **(P6)**. The culture of a cluster can benefit firms by providing a strategic direction and social integration [23]. One interviewee stated that “*It is a numbers game. The more people who leave comfortable jobs to start companies, the more successes we are likely to have*” **(P16).**

It has been demonstrated that policy changes can impact academics' decisions to create a spin-out. For example, in Norway, when rules changed to enable universities to take a stake in the IP rather than the academic owning 100% led to a 50% drop in spin-outs and patents (87). Conversely, in the USA, after relaxing IP rules in universities and making it easier for academics and industry to use IP, patents issued increased by 15 between 1980 and 2003 (88).

“*If you leave a multinational to create a start-up, you will take a salary reduction. Enabling the investor to use a previous tax paid as a support during the first few years would encourage people to take the chance*” **(P16).**

The tax treatment of shares is a significant disincentive to leave multinationals and join a start-up. The tax becomes due on all shares at that vesting value when shares vest. It is common for the sale of a start-up that several rounds of share vest at separate times depending on milestones. For example, some vest immediately, the second group at a regulatory milestone and a third at sales achievements several years in the future. As one individual stated “*Tax becomes due on all the shares at the first milestone, yet the individual cannot sell or realise the value of the shares, and they may never be worth the value of the tax paid. Tax should become liable when the value of shares is realised*” **(P16).**

Small companies can use employee share ownership programs (ESOPs) as an incentive to attract and retain employees. However, tax falling due before the value can be realised means this is not working for IDEs in Ireland. One interviewee commented in relation to this that “*some imagination on the tax treatment of ESOPs would significantly help entrepreneurs attract talent*” **(P14)**.

Start-ups encourage a culture of differentiated innovation. In a compiled list of 32 start-ups in Galway, Ireland, just 10 are in similar therapy areas as the local multinationals with 69% of start-ups having introduced new therapies to the Galway cluster.

Where the start-ups did treat therapies that local multinationals have a presence in, the start-ups have a point of difference in treating a currently unmet clinical need. Examples of companies that have developed vascular treatments differentiated from local multinationals include: Embo Medical, which has a one-shot device to close blood flow in the vascular, and InVera Medsystem is developing a unique treatment for Chronic Venous Disease.

## 5 Discussion and implications for policy

This study had two main RQ's which were met in this study.

The medical device Industry in Ireland has been a significant success employing 42,000 people with exports of US$15B in 2021. In terms of the different components of an industrial culture, The Irish Medtech cluster is strong on infrastructure, human capital, culture, and funding (RQ1).

The cluster in Ireland aligns with the literature proves that competitiveness is very local and is often in a small geographic area [4]. As with other studies the success of the Galway cluster and the competitive advantage factors are partially due to the local culture and how connections are made between stakeholders [4,9]. As Porter [2] stated that the critical thing is not a location's competitive factors but the rate at which they can be created, upgraded and customised to particular industries. This has been the case in Irelands Medtech cluster where the cluster has evolved and become more sophisticated and developed specializations.

The second RQ investigated the opportunities to improve the Irish Medtech cluster's health in terms of its culture, collaboration, and future regarding human capital, funding, innovative products and practices, demand for products, good infrastructure, and culture. Strategies to improve industrial clusters should seek to align the local ecosystem requirements and national regulations. Analysis for improvements of the ecosystem should be completed with the local stakeholders. Entrepreneurs should be the key voice in forming the strategy to improve the ecosystem [28]. Cardiovascular and orthopedic devices make up 75% of Ireland medical device exports by value [14]. The cluster should continue to build on these successes and seek to brand itself as a location of excellence. For example, for cardiovascular, “lots of people have a little bit of Galway in their heart”.

There are proven frameworks that have been successful in creating and developing clusters. Examples of these are the MIT Regional Economic Accelerator Program and the Boulder Thesis. Adapting a proven framework will give the best chance of success. This study puts forward a series of recommendations for the Irish Medtech cluster based on the literature review and qualitative research. Table 7 outlines a summary of the results and recommendations.

**Table 7:** Summary of Results and Recommendations (Source: Authors own work).

Area	Issues	Recommendation
Human Capital	Demand for labour has run ahead of the supply of labour. Expensive to get advice from specialists such as legal, intellectual property and regulatory viewpoint for start-ups. Desirable to increase the rate of spin-outs and patents from universities. Reduce equity stakes universities seek for IP—today's standard is 10–15%.	Continue reviewing educational courses and add to specialist courses as required. Create a pool of experts that are available to advise early-stage start-ups. Grant academics 100% rights on research they have completed. This has been shown to double the number of spin-outs. Reduce university stakes requested in return for IP to 5%. Again, this would be among the lowest levels globally.
Funding	Initial valuations by EI overvalue companies and make later rounds difficult to fund. EI demand preference shares so they get paid if the company closes. Research is well-funded for companies that are established. Shortage of seed funding. Tax treatment of shares and ESOPs are a significantly negative incentive for entrepreneurial activity.	EI be prepared to take a larger dilution to reflect the company's value. EI takes the shares on the same terms as others. Move more resources to translational research. Create a national fund. Encourage tax incentives for people to invest their money. The money would be focused on seed funding. Change tax rules on shares so tax is due only when shares are sold. This will make it easier to attract staff.
Infrastructure	Poor access to complete clinical trials locally. Difficult to get approval for clinical trials Few facilities to complete large animal trials. The cluster is missing accelerator programmes that are common in USA and Israel. Capacity is restricted. Not enough physical space to facilitate start-ups. Ireland's R&D is underdeveloped; potential for an extra €750 m spent.	Establish clinical infrastructure and policies to support. This includes physical space, dedicated personnel, specialist centres, standard systems, and expectations. Change policies to enable simple approval, an ideal situation being specific approvals for individual devices to an individual patient. Include HPRA in policies. Establish a lab capable of animal studies. Combined with a veterinary college would be ideal. Copy working methods of successful accelerators in the USA, for example, Jlabs or Northwell. Increase investment in facilities that are shown to work, I hubs Galway, Bio Innovate and similar facilities in Ireland. The actions above on clinical trials would help attract more R&D capacity to the region.
Demand	Small home markets. The cluster is focused on a few narrow therapeutic areas. Long lead time to create new products. There are capacity constraints in providing materials and services for the industry. Examples of constraints shared by interviewees are sterilisation services and specialists' materials, for example, high-performance polymers (Polyimide) causing constraints in subcontract manufacturing. Difficult to bring new products to market due to regulatory requirements. Results in unmet clinical needs, late bedside patient access, and a less competitive market. Although the medical device market is growing, the average price reduces yearly.	Continue born global strategy for companies; the priority market should currently be the USA. Continue development of start-up ecosystem. Start-ups are more likely to develop differentiated products. Look at class 1 products with a shorter time to market. Multinationals identify strategic suppliers not in the region. Then, these companies can be targeted to move to the region. Lobby to change regulations mimic the approach used in the USA that accelerates approval for innovative De novo products. Establish a formal alliance to lobby that includes local regulatory bodies. Constantly renew portfolio with differentiated products that attract a premium. (Vibrant start-up ecosystem required for success).
Culture & Incentives	Underdeveloped identity of Galway Hub. Significant funds are spent on research in universities. Seek to have translational research to turn it into commercial opportunities. The primary measure in university research is the papers published. Most research is not made commercial. It's been shown that there is more commercial success and a doubling of spin-outs if the inventor can access IP for free. NDAs (Non-Disclosure Agreements) restrict the permeability of the university.	Brand and establish identity. Set the expected behaviours of collaboration and cooperation. Use programs like SPARK, as used at Stanford, for translational work. Give credit for work to commercialise research. For example, give credit for well-researched business plans. Have mentors for research that can guide potential commercial ideas. Offer free licenses to the inventor of IP in Universities if used within 2 years. Reduce restrictions on NDAs and have no limitation on contacting or hiring staff from the university in NDA.

## 6 Conclusions

Strategies can be created and applied to shape industrial clusters. The evolution of the Irish Medical device cluster is an example of successful evolving strategy implementation. Weaknesses of funding in the 1990s have been significantly improved, enabling entrepreneurial activity and a healthy medical device start-up cluster to be established. Due to declining sales prices and rising labour costs, the cluster must continually reinvent itself. Analysis of the cluster demonstrates the cluster's weaknesses and highlights its opportunities.

This study has implications for the Medtech industry and the wider Irish economies health. The cluster has proven resilient with consistent growth, but there is the potential for more; it can unleash €700 million in extra research spend and increase the number of innovation-driven enterprises. This will provide economic benefits while improving patient outcomes on a global scale. Policy changes and funding can directly impact the culture, increasing entrepreneurial activity. Reduced equity stakes from universities for IP can increase the number of indigenous spinouts directly. Implementing a strategy that while difficult as it requires the alignment of many strands, including financial, regulatory, incentives, clinical infrastructure, and improved collaboration between stakeholders has implications for the successful health of the cluster. A coherent strategy while difficult to implement but will create a competitive advantage that is hard for other regions and countries to copy. From an academic implication viewpoint, the study demonstrates how a cluster has evolved and become successful and can be utilised to educate others as well as show how academia can aid in development and success of startups. From an industry viewpoint this study demonstrates the opportunities to improve the success of the health and can be leveraged by government organisations to aid policy to develop the cluster.

This study is the first of its kind to study the health of a cluster and offer recommendations for its future success via key elements that make up the cluster.

A limitation of this study is that it is based on a single case study that of the Irish Medtech cluster however the learnings are generalizable for other clusters.

Future research opportunities are to investigate the gap in clinical trials supports and how that can be overcome as well as longitudinal case studies on startup spin off enterprises to ascertain their specific challenges.

## Disclosure statement

The authors report there are no competing interests to declare.

## CRediT authorship contribution statement

**David McKernan:** Conceptualization, Formal analysis, Investigation, Visualization, Writing – original draft. **Olivia McDermott:** Conceptualization, Investigation, Project administration, Supervision, Writing – original draft, Writing – review & editing.

## Declaration of competing interest

The authors declare the following financial interests/personal relationships which may be considered as potential competing interests:Olivia McDermott reports article publishing charges was provided by University of Galway. Olivia McDermott reports a relationship with University of Galway that includes: employment. n/a.
